# Toward an Optimized Staging System for Pancreatic Ductal Adenocarcinoma: A Clinically Interpretable, Artificial Intelligence–Based Model

**DOI:** 10.1200/CCI.21.00001

**Published:** 2021-12-22

**Authors:** Dimitris Bertsimas, Georgios Antonios Margonis, Yifei Huang, Nikolaos Andreatos, Holly Wiberg, Yu Ma, Caitlin Mcintyre, Alessandra Pulvirenti, Doris Wagner, J. L. van Dam, Francesca Gavazzi, Stefan Buettner, Katsunori Imai, Georgios Stasinos, Jin He, Carsten Kamphues, Katharina Beyer, Hendrik Seeliger, Matthew J. Weiss, Martin Kreis, John L. Cameron, Alice C. Wei, Peter Kornprat, Hideo Baba, Bas Groot Koerkamp, Alessandro Zerbi, Michael D'Angelica, Christopher L. Wolfgang

**Affiliations:** ^1^ Operations Research Center, Massachusetts Institute of Technology, Cambridge, MA; ^2^ Department of Surgery, Memorial Sloan Kettering Cancer Center, New York, NY; ^3^ Department of Surgery, Johns Hopkins University School of Medicine, Baltimore, MD; ^4^ Department of Internal Medicine and Taussig Cancer Institute, Cleveland Clinic, Cleveland, OH; ^5^ Section of Pancreatic Surgery, Humanitas Clinical and Research Center-IRCCS, Milan, Italy; ^6^ Department of General Surgery, Medical University of Graz, Graz, Austria; ^7^ Department of Surgery, Erasmus MC, University Medical Center, Rotterdam, the Netherlands; ^8^ Department of Gastroenterological Surgery, Graduate School of Life Sciences, Kumamoto University, Kumamoto, Japan; ^9^ Technical Chamber of Greece (TEE-TCG), Athens, Greece; ^10^ Department of General, Visceral and Vascular Surgery, Charité Campus Benjamin Franklin, Berlin, Germany; ^11^ Department of Surgery, Northwell Health, Manhasset, NY

## Abstract

The American Joint Committee on Cancer (AJCC) eighth edition schema for pancreatic ductal adenocarcinoma treats T and N stage as independent factors and uses positive lymph nodes (PLNs) to define N stage, despite data favoring lymph node ratio (LNR). We used artificial intelligence–based techniques to compare PLN with LNR and investigate interactions between tumor size and nodal status.

**METHODS:**

Patients who underwent pancreatic ductal adenocarcinoma resection between 2000 and 2017 at six institutions were identified. LNR and PLN were compared through shapley additive explanations (SHAP) analysis, with the best predictor used to define nodal status. We trained optimal classification trees (OCTs) to predict 1-year and 3-year risk of death, incorporating only tumor size and nodal status as variables. The OCTs were compared with the AJCC schema and similarly trained XGBoost models. Variable interactions were explored via SHAP.

**RESULTS:**

Two thousand eight hundred seventy-four patients comprised the derivation and 1,231 the validation cohort. SHAP identified LNR as a superior predictor. The OCTs outperformed the AJCC schema in the derivation and validation cohorts (1-year area under the curve: 0.681 *v* 0.603; 0.638 *v* 0.586, 3-year area under the curve: 0.682 *v* 0.639; 0.675 *v* 0.647, respectively) and performed comparably with the XGBoost models. We identified interactions between LNR and tumor size, suggesting that a negative prognostic factor partially overrides the effect of a concurrent favorable factor.

**CONCLUSION:**

Our findings highlight the superiority of LNR and the importance of interactions between tumor size and nodal status. These results and the potential of the OCT methodology to combine them into a powerful, visually interpretable model can help inform future staging systems.

## INTRODUCTION

The American Joint Committee on Cancer (AJCC) TNM staging system remains the gold standard for prognostication in pancreatic ductal adenocarcinoma (PDAC). Three main criteria have been proposed to evaluate how well the TNM staging system fulfills its mission^1^: reproducibility, simplicity, and discriminatory ability. Striving for simplicity, however, involves an implicit trade-off with discriminatory ability, as the addition of more prognostic variables has been shown to lead to modest gains in the latter, but at the cost of increased model complexity. The compromise approach followed in AJCC staging systems and clinical practice to date tends to favor simplicity; as a result, the area under the curve (AUC) of both the AJCC seventh and eighth edition staging systems for PDAC remains quite low (approximately 0.6), reflecting suboptimal discriminatory ability.^1,2^

**CONTEXT**

**Key Objective**
To our knowledge, this work marks the first successful use of artificial intelligence and game theory to modify an American Joint Committee on Cancer classification system not only among patients with pancreatic ductal adenocarcinoma but also in oncology as a whole. This novel approach to constructing staging systems may have significant future applications across different malignancies.
**Knowledge Generated**
This study contradicts the assumption that T and N components of the TNM staging system are independent of each other, by showing that they exhibit clinically important interactions. These interactions proved to be prognostically important as they leverage increased prognostic power without hindering clinical interpretation.
**Relevance**
Our findings may help resolve the long-standing debate regarding the optimal proxy of nodal status in pancreatic ductal adenocarcinoma, whereas they suggest that for a given patient, the beneficial impact on prognosis of low T or N stage may be overridden by that of coexisting high N or T stage to a greater extent than would be expected by simple addition.

Increasing the discriminatory ability of the AJCC eighth edition without introducing new factors or unduly complicating the model is evidently a daunting challenge. One promising approach is to define the N component on the basis of the ratio of positive lymph nodes divided by the total number of harvested lymph nodes (LNR) rather than the number of positive lymph nodes (PLNs) alone, which is the current practice.^3-5^ A second and possibly more widely applicable approach would be to stop considering the different components of the AJCC system as independent, additive prognostic factors, but to assess the possibility of interactions between them that may render the prognostic effect of T stage dependent on the respective N stage and vice versa (eg, it is possible that low T stage does not confer the same beneficial prognostic impact in patients with a high burden of nodal metastases compared with those without nodal involvement). In turn, if such interactions exist and are significant, their incorporation into the model can increase discriminatory power without the need to include additional prognostic factors.

We first assessed the relative prognostic power of LNR and PLN via the novel shapley additive explanations (SHAP) methodology, which uses mathematical techniques used in game theory to estimate a variable's contribution to a prognostic model.^6^ After selecting the more promising of these two predictors to reflect nodal status and incorporating tumor size as an additional variable, we developed a prognostic model with the aid of optimal classification trees (OCTs), an artificial intelligence–based methodology that can readily capture interactions between variables and present them in a visually interpretable manner.^7^ The OCT model was subsequently compared with the AJCC eighth edition staging system and XGBoost, a state-of-the-art, but less interpretable, machine learning approach.

## METHODS

### Patient Selection

All patients who underwent a partial or total pancreatectomy for PDAC as part of routine clinical care between January 1, 2000, and December 31, 2017, at Johns Hopkins Hospital, Memorial Sloan Kettering Cancer Center, Erasmus Medical Center, Fondazione IRCCS Istituto Nazionale Dei Tumori, Graz University, and Kumamoto University were retrospectively identified from institutional databases. The study was conducted in accordance with the ethical standards of the participating institutions and was approved by their institutional review boards. A detailed ethics statement is provided in the Data Supplement.

Patients with metastatic disease were excluded. We also excluded patients with grossly positive resection margins (as determined by review of the operative and pathology reports and clinical documentation authored by the attending surgeon) because the presence of macroscopic residual disease precludes accurate assessment of tumor size on pathology.^8^ Similarly, patients treated with neoadjuvant therapy were excluded because of the lack of consensus definitions on how to assess gross tumor size after response to treatment.^9^ Patients who died within 30 days of resection and those with missing data on survival outcomes, tumor size, or nodal status (as determined by pathologic examination of the resected specimen) were also excluded.

### Data Extraction and Study Outcomes

We collected data on clinicopathologic variables with the aid of a centrally designed data report form that was used by all participating institutions (Table 1). All pathologic data were derived from the available pathology reports. All clinical and pathologic variables necessary to estimate AJCC stage for each patient were included in the respective data report form. Staging was subsequently determined centrally by the Johns Hopkins Hospital investigators. We used state-of-the-art nonlinear imputation methods to help replace missing entries with artificially created substitute values that aim to approximate the variable distribution that would be observed in a complete data set.^10^ Resections were considered margin positive when tumor cells were found at the margin or within 1 mm of the margin, as defined by the Royal College of Pathologists in 2017.^8^ T, N, and M stages were defined on the basis of the eighth edition of the respective AJCC staging manual. Overall survival was calculated from the date of surgery to the date of death or last follow-up. The process followed by each institution to determine survival outcomes is presented in the Data Supplement.

**TABLE 1.**
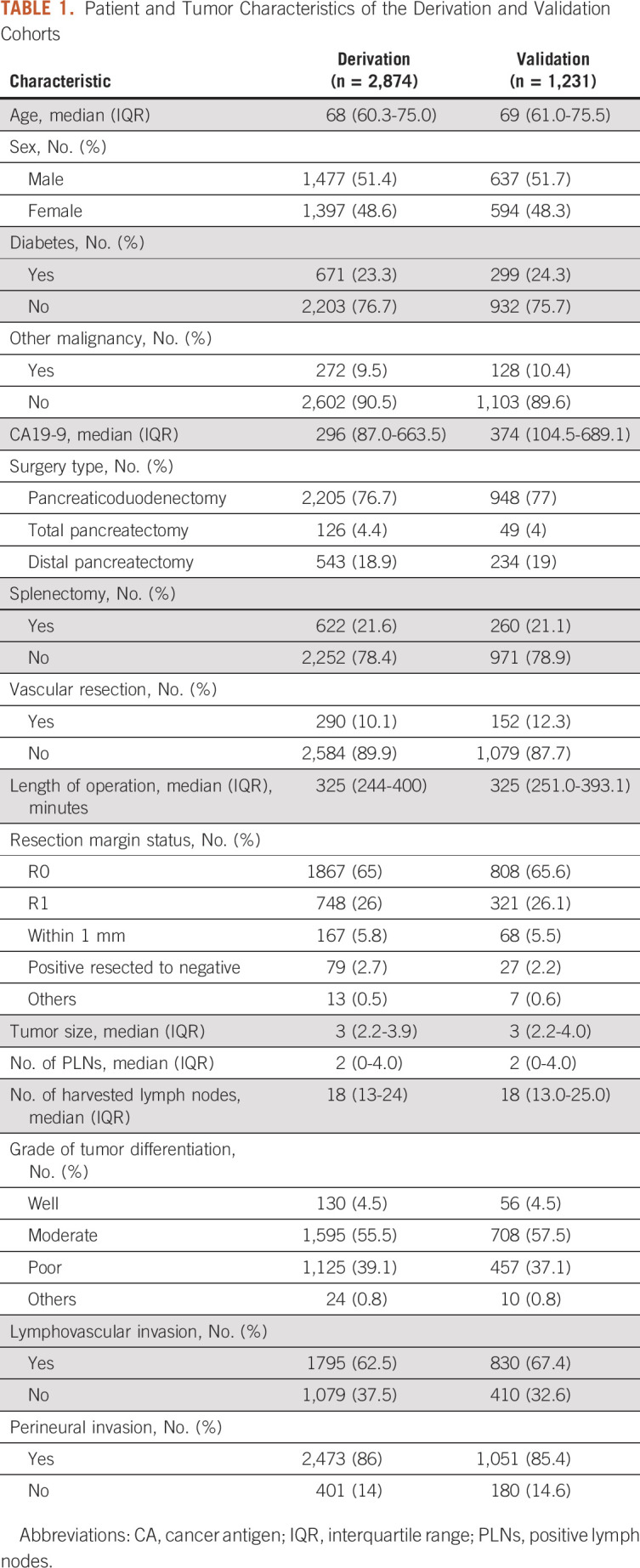
Patient and Tumor Characteristics of the Derivation and Validation Cohorts

### XGBoost and SHAP Analysis

To discern whether LNR or PLN is the stronger predictor of survival outcomes and, consequently, the best basis for defining nodal status, we trained two separate XGBoost models (probability of death was the outcome of interest) at the 1-year and 3-year time points with the aid of multiple prognostic factors (including both PLN and LNR) and subsequently used SHAP analysis to determine the relative importance of these predictors. Further details regarding XGBoost and SHAP analysis can be found in the Data Supplement. The magnitude of a given SHAP value reflects the importance of the predictor in question.

To illustrate the results of the SHAP analysis, we constructed variable importance plots that list the predictors incorporated in each XGBoost model on the basis of the respective SHAP values.

### Estimating Interactions Between Predictors With the SHAP Model

To better assess the interplay of tumor size and nodal status, we used SHAP on the XGBoost models derived above and plotted the respective interaction values for a range of different predictor values for the 1-year and 3-year time points. Further details regarding how the SHAP method can help assess interactions between predictors can be found in the Data Supplement.

### Developing the OCT Models

The selection of the OCT method to develop the final prognostic models is explained in the Data Supplement. Separate OCTs were trained to predict 1-year and 3-year death rates; outcomes at each time point were generated using the following principles: (1) patients who died before the time point of interest were coded as dead, (2) patients who died after the time point of interest were coded as alive, (3) patients who were censored before the time point of interest because they were lost to follow-up were excluded, and (4) patients who were censored after the time point of interest were coded as alive.

To develop the OCTs, the cohort was randomly divided into derivation (70%) and validation (30%) data sets. To limit overfitting, the OCTs were tuned with the use of the following two hyperparameters: maximum depth of tree and minimum number of samples per leaf. Grid search and cross-validation were used to help us select hyperparameters.

### Model Evaluation

We compared the AUC of the 1-year and 3-year OCTs with that of the AJCC eighth edition staging system in both the derivation and validation data sets. To further assess the performance of the OCT methodology, we trained additional 1-year and 3-year XGBoost models on the basis of the same factors as the OCTs for the purpose of comparison. Details regarding computing can be found in the Data Supplement.

## RESULTS

### Patient Characteristics and Survival Analysis

**FIG A1.**
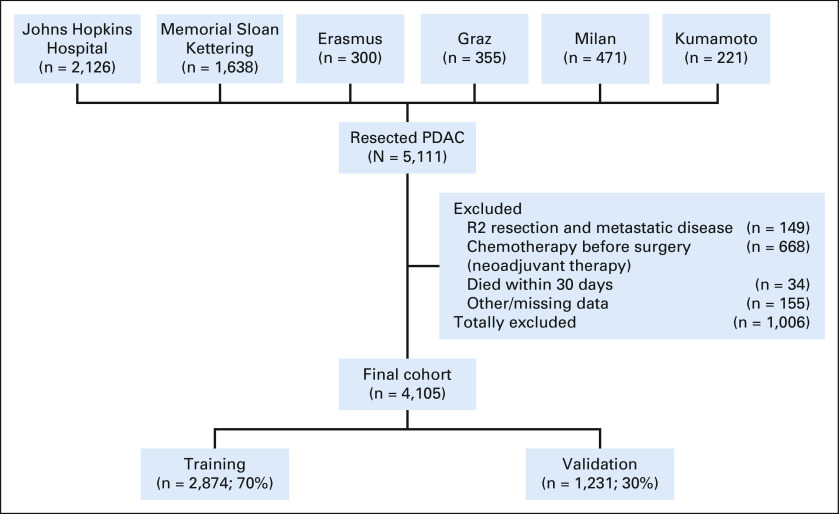
Flowchart of the study cohort. PDAC, pancreatic ductal adenocarcinoma.

The final study population included 4,105 patients (Appendix Fig A1). Of those, 2,874 patients (70%) comprised the derivation cohort and 1,231 patients (30%) the validation cohort. Detailed clinicopathologic characteristics of the two cohorts are presented in Table 1; participating institutions were represented in proportion to their contribution to the overall study population (Appendix Table A1). The distribution of disease stages in the derivation and validation cohorts according to the AJCC eighth edition staging system is presented in Appendix Table A2. The proportion of cases with missing values in each variable is provided in Appendix Table A3. A total of 508 patients were censored before the 3-year time point; their characteristics in comparison with the remaining cohort are presented in Appendix Table A4. As can be seen, the two groups were well-balanced overall and any observed differences were generally of small magnitude.

In the derivation cohort, the 1-year and 3-year death rates were 25.7% and 71.4%, respectively. In the validation cohort, the 1-year and 3-year death rates were 24.8% and 71%, respectively.

### SHAP Variable Importance Plot

**FIG A2.**
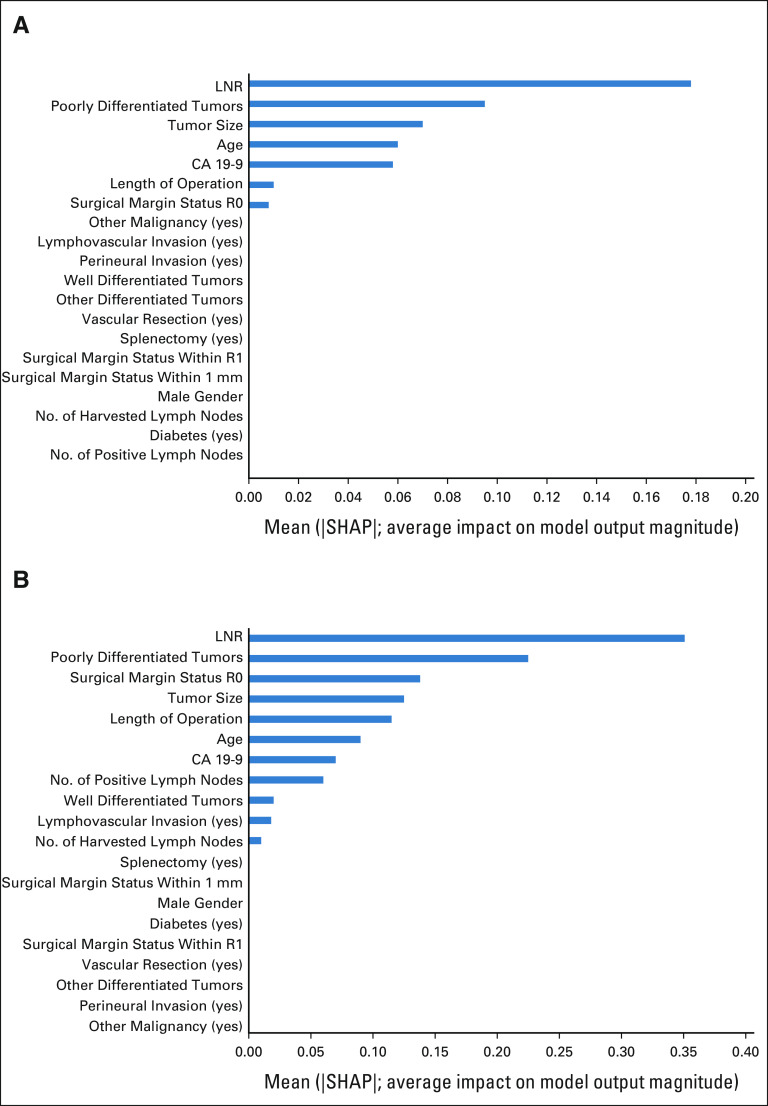
SHAP variable importance plot: (A) year 1 and (B) year 3. CA, cancer antigen; LNR, lymph node ratio; SHAP, shapley additive explanations.

According to the SHAP analysis of the XGBoost models (Appendix Fig A2), the mean absolute SHAP value of LNR was much higher than that of PLN at both time points, indicating that LNR is a stronger predictor. As such, we incorporated LNR rather than PLN as a proxy of nodal status in the 1-year and 3-year OCTs.

### 1-Year and 3-Year OCTs

**FIG 1.**
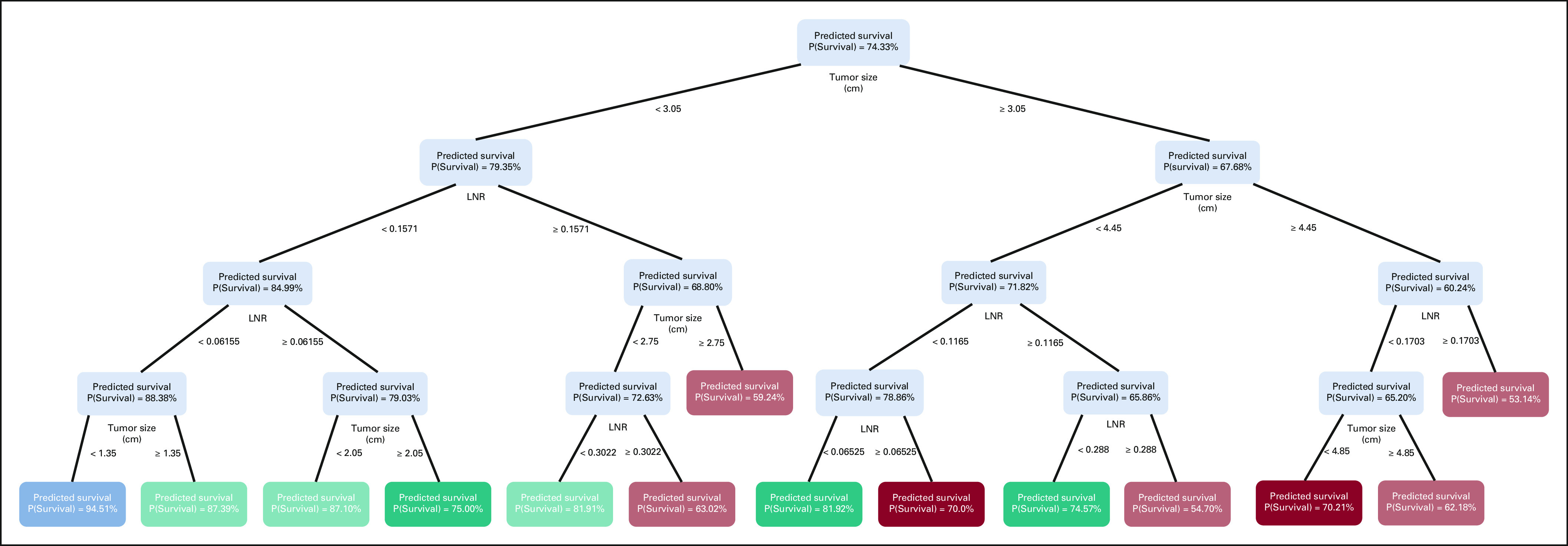
Prediction of 1-year overall survival with the aid of optimal classification trees. LNR, lymph node ratio.

**FIG 2.**
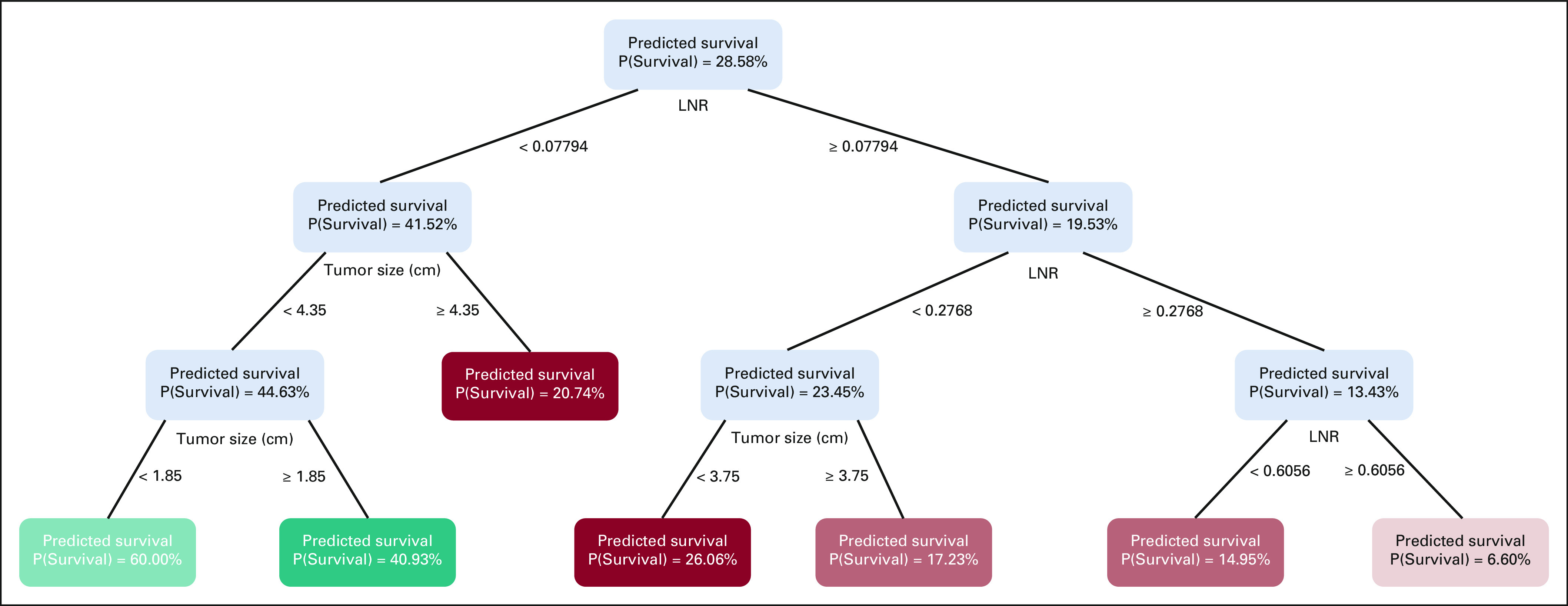
Prediction of 3-year overall survival with the aid of optimal classification trees. LNR, lymph node ratio.

The OCTs for predicting death within 1 and 3 years of surgery are illustrated in Figures 1 and 2 and in Ref. 11. The 1-year and 3-year overall survival estimates for a specific patient can be derived with the aid of the online calculator.^11^

### OCT Performance and Comparison With the AJCC Eighth Edition Staging System and the XGBoost Model

In both the derivation and validation cohorts, the AUC of the OCT compared favorably with that of the AJCC eighth edition staging system at the 1-year and 3-year time points (Table 2). The OCTs slightly underperformed the XGBoost model in both the derivation and validation cohorts at the 1-year and 3-year time points (Table 2). Subgroup analyses restricted to patients with a low (< 15 lymph nodes) or a high (≥ 15 lymph nodes) number of harvested lymph nodes yielded similar results. Specifically, in the validation cohort, the AUC of the 1-year OCT increased to 0.647 (*v* 0.638), whereas the AUC of the 3-year OCT increased to 0.698 (*v* 0.675).

**TABLE 2.**
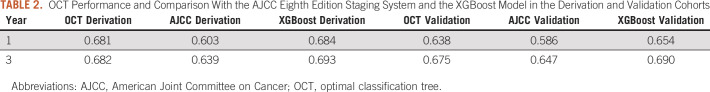
OCT Performance and Comparison With the AJCC Eighth Edition Staging System and the XGBoost Model in the Derivation and Validation Cohorts

### SHAP Interaction Values

**FIG 3.**
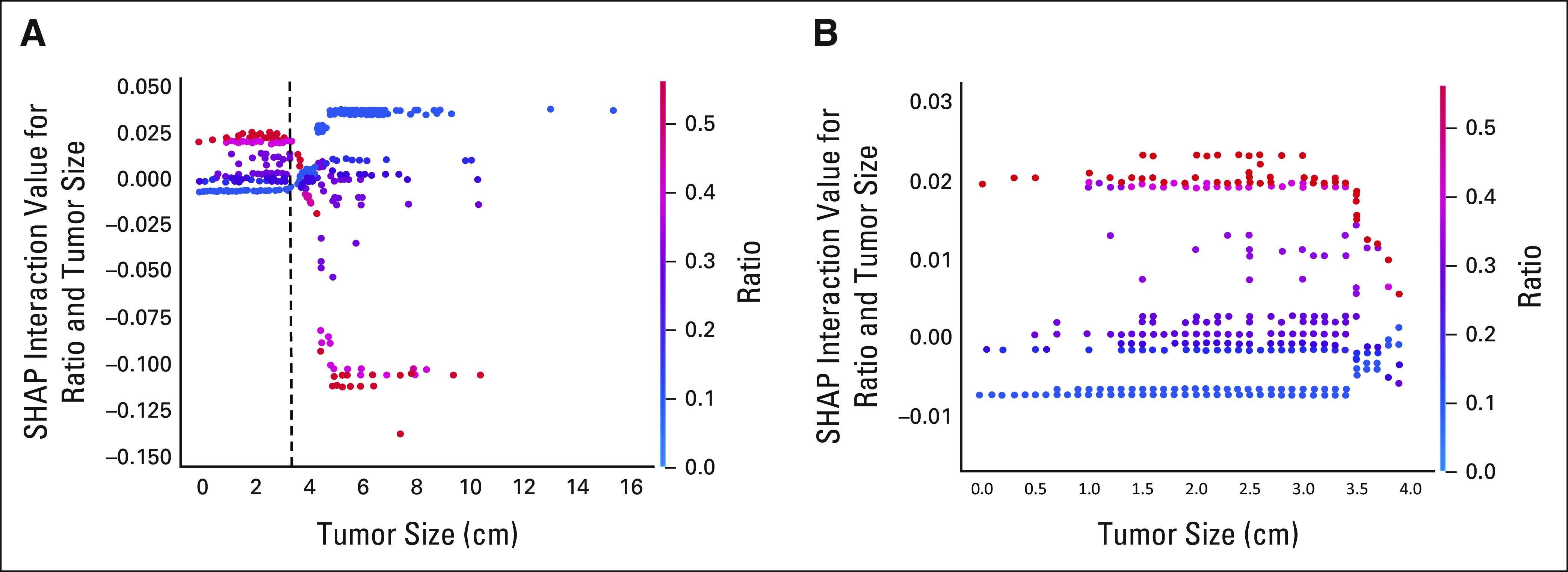
SHAP interaction values for different tumor sizes and lymph node ratios at the 1-year time point. (A) Full size; (B) zoom in of figure part (A). SHAP, shapley additive explanations.

**FIG 4.**
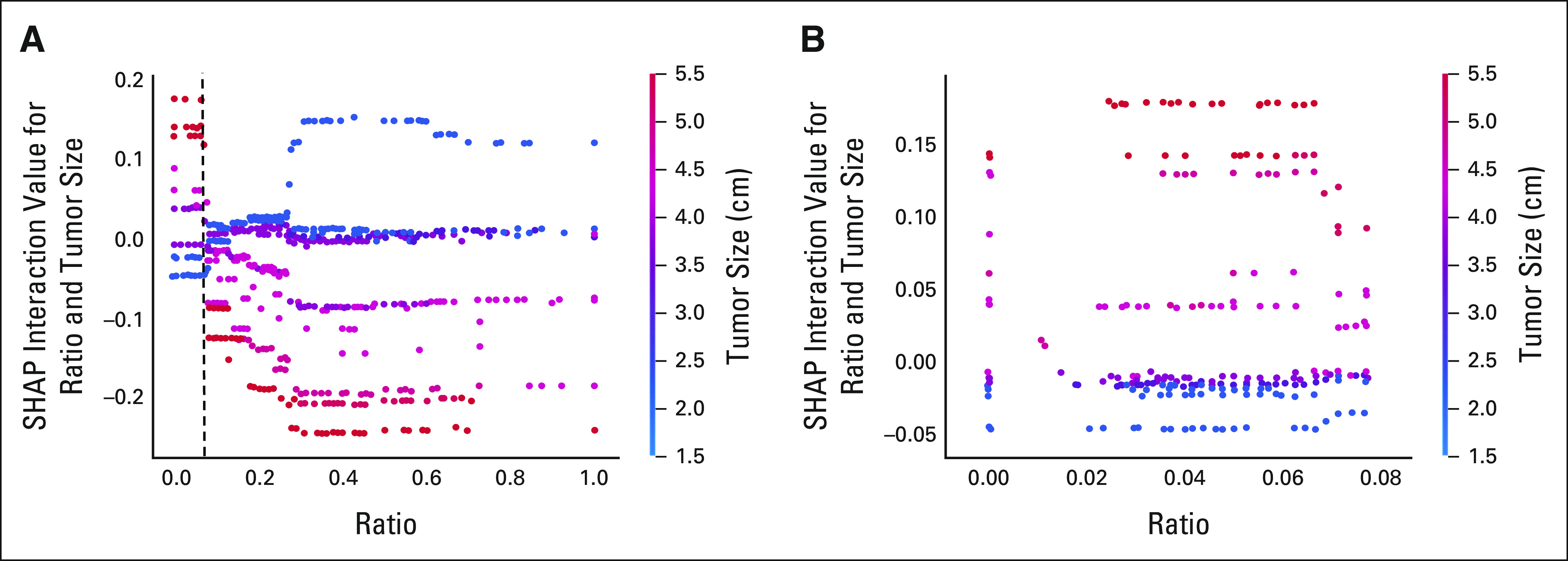
SHAP interaction values for different tumor sizes and lymph node ratios at the 3-year time point. (A) Full size; (B) zoom in of figure part (A). SHAP, shapley additive explanations.

With the aid of SHAP, we analyzed the feature interactions in the full XGBoost model (used to determine whether LNR or PLN is the stronger predictor as discussed above) to provide a data-driven validation of the OCT cutoffs. The results of this analysis for the 1-year and 3-year time points are depicted in Figures 3 and 4, respectively. Further details regarding the explanation of Figures 3 and 4 can be found in the Data Supplement.

## DISCUSSION

The relative prognostic value of LNR and PLN has been debated for a long time; although most studies appear to support LNR as a more potent predictor, consensus on this point is lacking.^12-15^ This might partly explain why LNR has not been incorporated into the AJCC staging system. Our analysis, performed with the aid of SHAP in a very large data set, clearly demonstrated that LNR was not only superior to PLN but also the most important of all commonly used clinicopathologic predictors at both time points. This is consistent with a recent study that reported that the presence of nodal disease was the most important among several predictors in a large PDAC cohort.^16^ The prognostic value of LNR can likely be attributed to its ability to limit nodal disease misclassification and more accurately reflect tumor burden. It is also theoretically possible that LNR more closely reflects the adequacy of nodal resection, which, in turn, may be tied to outcomes. These findings may both help resolve the ongoing debate and pave the way for the use of LNR in future AJCC editions. We used Optimal Imputation to account for missing values in secondary prognostic variables. Although this did not affect the main analyses, which relied on variables that were not imputed, the comparison of PLN and LNR was based on a multivariable model and might have been indirectly affected by this approach. However, as the available data were reasonably complete for the majority of important predictors, any corresponding impact was likely limited.

After deciding in favor of LNR as a proxy of nodal status, we proceeded to train 1-year and 3-year OCTs and assess their discriminatory ability. In both the derivation and validation cohorts, the AUC of the present model compared favorably with that of the AJCC eighth edition staging system at the 1-year and 3-year time points and surpassed that of previous validations of the AJCC eighth edition reported in the literature.^2,8^ The prognostic advantage of our model over the AJCC staging system was retained even among patients with accurate nodal staging (defined as ≥ 15 harvested nodes) in whom the benefit of using LNR rather than PLN may be less pronounced. In fact, the AUC of the 3-year OCT in this patient group was nearly 0.7, which is among the highest ever reported in the literature.

The OCT method has another important advantage over the AJCC schema, as it considers tumor size and nodal status concurrently, thus capturing underlying interactions between them; on the contrary, prognostic cutoffs in the AJCC staging system are developed independently for each variable. Interestingly, the 3-year OCT used essentially the same tumor size cutoffs as the AJCC eighth edition: 1.85 cm (AJCC T1: tumor size < 2 cm), 1.85-4.35 cm (AJCC T2: 2 cm < tumor size ≤ 4 cm), and ≥ 4.35 cm (AJCC T3: tumor size > 4 cm). As these cutoffs were selected in an unbiased way after considering tumor size as a continuous variable, the resulting level of consistency is remarkable.

To further assess the internal validity of the cutoffs identified by the OCTs, we performed an analysis of interactions with the aid of the SHAP method in independently devised XGBoost prognostic models. Importantly, the same cutoffs were identified with the use of this alternative methodology. In the case of 1-year risk of death, a tumor size of 3.05 cm was not only the first point of divergence for the respective OCT but also the point where the interactions between LNR and tumor size switched direction, thus indirectly demonstrating the poor prognostic consequences of exceeding that tumor size, as discussed above. Similar results were noted for LNR at the 3-year time point. Taken together, the identification of the same cutoffs by two distinct mathematical methods not only illuminates the significance of the underlying variable interactions but also supports the overall reliability of the findings.

Beyond simply validating prognostic cutoffs, the SHAP method allowed us to explore the interactions of tumor size and LNR as continuous variables for the first time. Importantly, the results suggest that the combination of large tumor size (as determined by the above cutoff) with minimal nodal involvement leads to worse prognosis than would be expected by aggregating the impact of the two individual factors; a similar pattern is observed for patients with a LNR ≥ 0.08 and small tumors. This implies that the adverse prognostic effect of large tumor size or high LNR partly overrides the beneficial effect of low LNR or small tumor size, respectively. These results underline the importance of considering tumor size and nodal status concurrently.

The study has several limitations. The inclusion of multi-institutional data has introduced some heterogeneity; for example, there may be differences in surgical procedures and pathologic examination across centers that could theoretically result in variable lymph node yield or other discrepancies in reported pathologic variables. Moreover, outcome data are derived from patients who were successfully followed for a significant period of time, whereas patients who could not maintain follow-up until the time point of interest were censored and thus effectively excluded from the analysis. Although the two groups appear comparable on the basis of the available data, the possibility of underlying differences in unmeasured characteristics that could have affected the results cannot be entirely excluded. Finally, although available data on adjuvant chemotherapy administration are concordant with previous reports, detailed information regarding this aspect of care was not available in some institutional databases. Although this limits our ability to accurately estimate the percentage of patients treated with adjuvant chemotherapy, prescription practices at these two institutions are unlikely to have diverged substantially from the respective standards of care for the period. In turn, the positive impact of adjuvant chemotherapy on survival is likely reflected in our results, a fact that should be considered when using the model for prognostication.

In conclusion, we demonstrated the superiority of LNR over PLN as a prognostic factor and, subsequently, generated and validated a new prognostic model for patients with PDAC that incorporates interactions between tumor size and nodal status with the aid of the OCT methodology. Importantly, the resulting model comfortably outperformed the AJCC eighth edition staging schema and performed comparably with the state-of-the-art XGBoost model without sacrificing interpretability. Although the inclusion of additional predictors would have improved the model's AUC, it would have also precluded direct comparison with the AJCC schema and complicated the interpretation of interactions, thus frustrating the principal aims of the study. Additional performance optimization on the basis of a multivariable OCT model is feasible and should be evaluated in future reports. The resulting prognostic cutoffs identified by the model are highly original as they were derived by simultaneously considering tumor size in the context of nodal status and vice versa. Although external validation in patients from different geographic areas will have to await future studies, a supplementary analysis performed with the aid of the SHAP method confirmed the internal validity of our findings. Finally, the observed variable interactions are more than an abstract mathematical concept aimed at enhancing AUC, but can be readily interpreted at a clinical level; our findings suggest that unfavorable tumor size has a tendency to override favorable nodal status and vice versa, leading to more guarded prognosis than would be expected by adding the prognostic impact of each individual factor. If these observations are confirmed, a strong case can be made in favor of incorporating LNR and considering variable interactions in future AJCC editions. Perhaps more importantly, the ability of artificial intelligence–based interaction analysis to provide a sophisticated but interpretable view of the interplay between clinicopathologic variables may in due course lead to widespread prognostic, predictive, and, ultimately, prescriptive applications across different tumor types.

## Figures and Tables

**TABLE A1.**
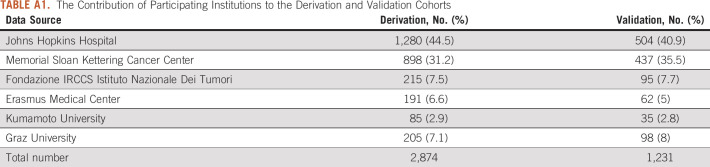
The Contribution of Participating Institutions to the Derivation and Validation Cohorts

**TABLE A2.**
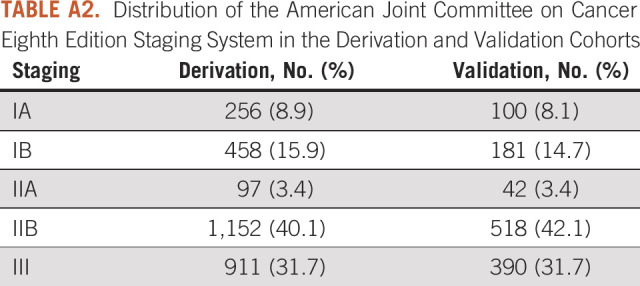
Distribution of the American Joint Committee on Cancer Eighth Edition Staging System in the Derivation and Validation Cohorts

**TABLE A3.**
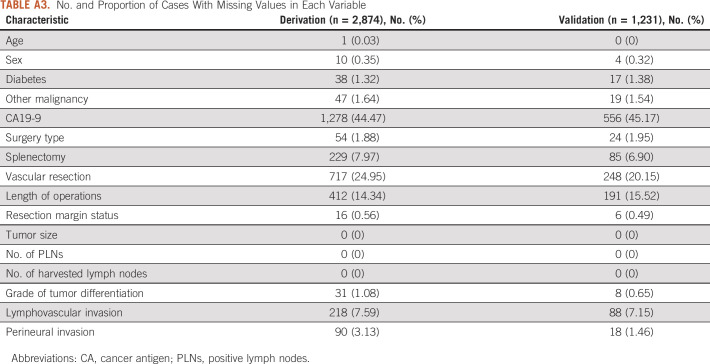
No. and Proportion of Cases With Missing Values in Each Variable

**TABLE A4.**
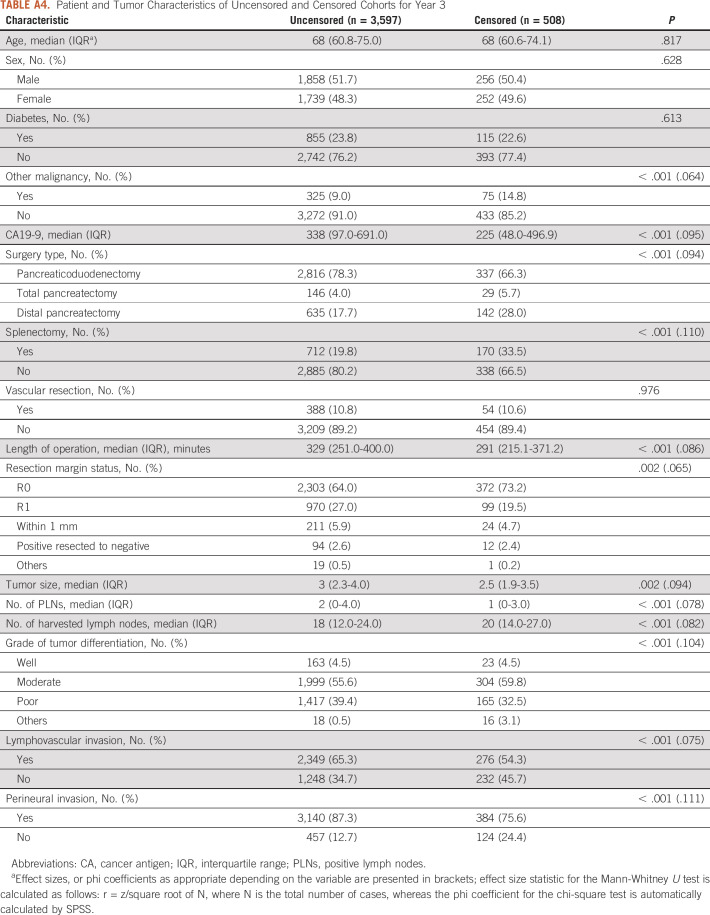
Patient and Tumor Characteristics of Uncensored and Censored Cohorts for Year 3
